# Supplementary material for: Neoadjuvant chemo-immunotherapy with camrelizumab plus nab-paclitaxel and cisplatin in resectable locally advanced squamous cell carcinoma of the head and neck: a pilot phase II trial
Source: Nat Commun. 2024 Mar 11;15:2177. doi: 10.1038/s41467-024-46444-z (PMC10928200; doi:10.1038/s41467-024-46444-z)
Supplement: Supplementary file 1 — Supplementary Information [file 41467_2024_46444_MOESM1_ESM.pdf]

Supplementary information

Content

Supplementary Table 1 Surgical complications……………………………………………3

Supplementary Figure 1 Presentative images of pre and post-treatment assessments………..4

Supplementary Figure 2 Swimmer plot of progression-free survival………………………5

Supplementary Figure 3 Distribution of TMB and PD-L1 expression level in patients with different anatomic sites and TNM stages……………………………………………………6

Supplementary Figure 4 Correlation analysis of radiographic tumor response with the density of tumor-infiltrating CD4+ T cells………………………………………………………7

Supplementary Figure 5 Correlation analysis between radiographic tumor regression and TMB, PD-L1 expression, or density of tumor-infiltering immune cells………………………8

Supplementary Figure 6 Comparisons of the density of tumor infiltration immune cells before and after neoadjuvant therapy in overall patients and those with pCR and non-pCR………..9

Supplementary Note Study Protocol……………………………………………………10

Supplementary Table 1 Surgical complications (n=27)

	Surgical complications, n (%)		
	**Grade 1**	**Grade 2**	**Grade 3**
Swelling, head and neck	5 (18.5)		
Postoperative hemorrhage	1 (3.7)	2 (7.4)	0
Pain, head and neck	2 (7.4)	0	0
Delayed wound healing	0	2 (7.4)	0
Numbness, head and neck	2 (7.4)	0	0
Intraoperative hemorrhage	1 (3.7)	0	0
Pneumonia	1 (3.7)	0	0
Pharyngeal fistula	0	0	1 (3.7)
Dysphagia	1 (3.7)	0	0

Supplementary Figure 1 Presentative images of pre and post-treatment assessments. CT images show an oropharyngeal patient with a CR (Pre: a; Post: b); while H&E images show an oral patient with a pCR (Pre: c; Post: d).

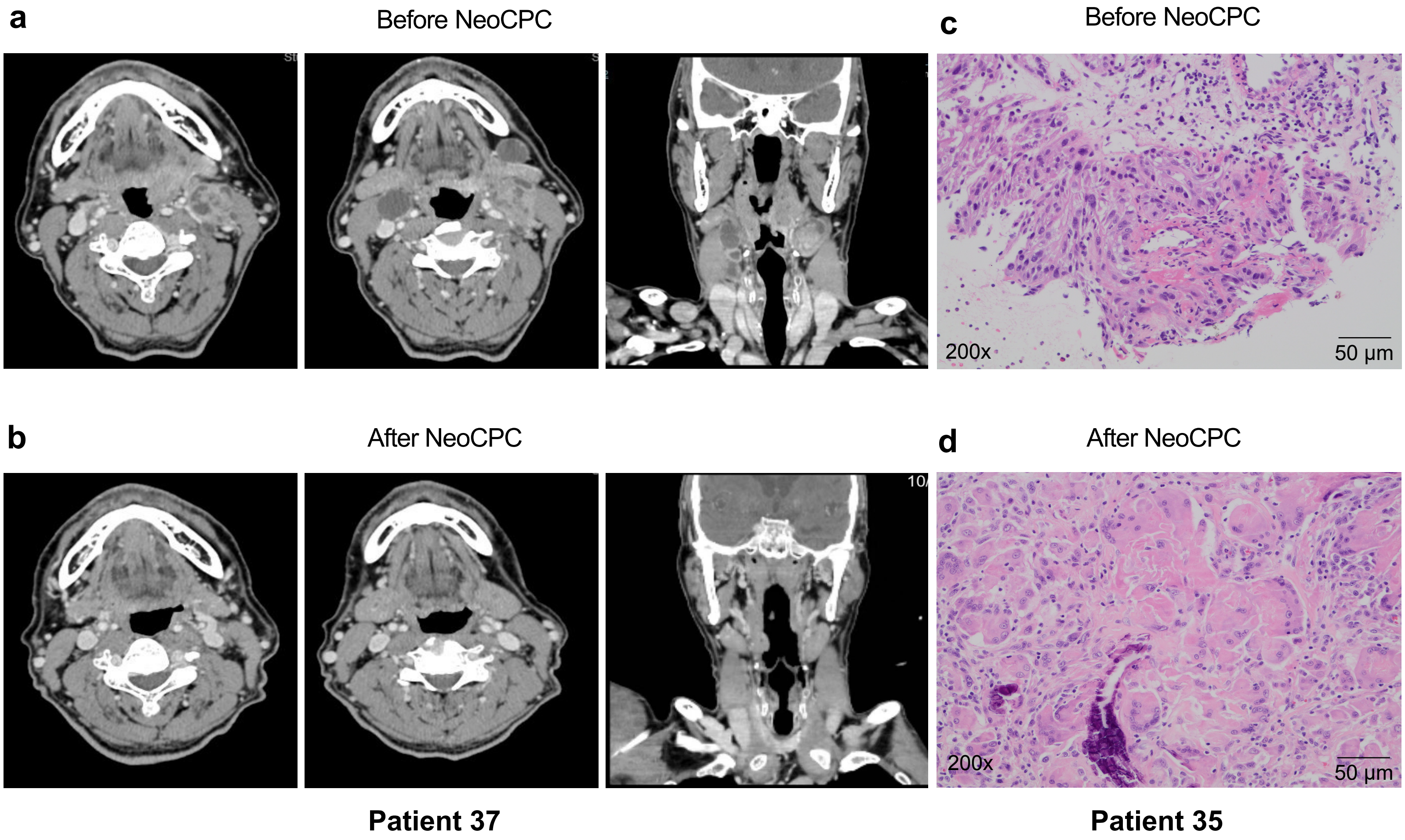

Supplementary Figure 2 Swimmer plot of progression-free survival (n=47). Source data are provided as a Source Data file.

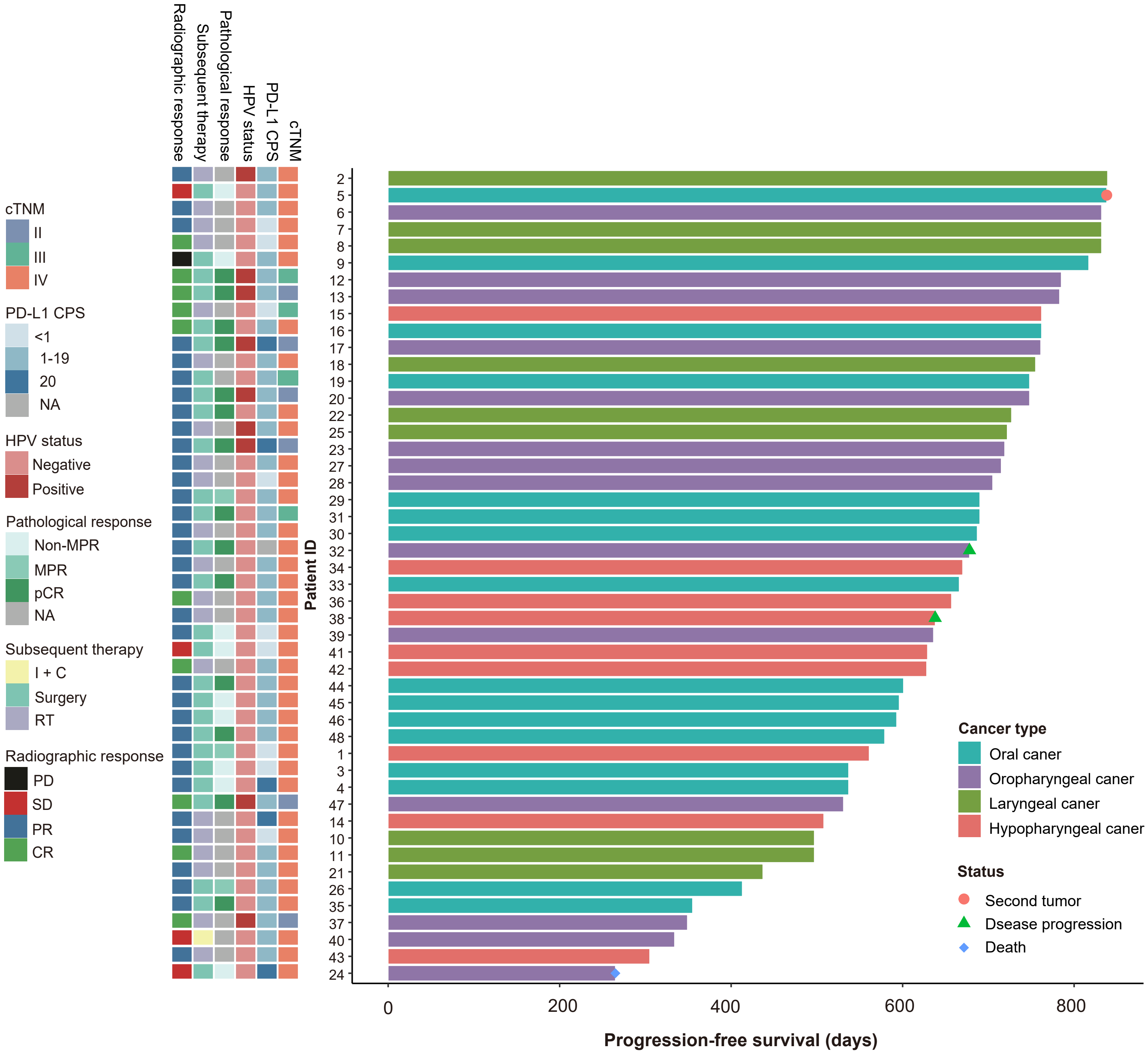

Supplementary Figure 3 Distribution of TMB and PD-L1 expression level in patients with different anatomic sites (a and c) and TNM stages (b and d) analyzed by using the Kruskal-Wallis test. The dot represents an individual data point. The center line with a circle represents mean value, and the bar represents standard deviation. OC: Oral cancer(n=16); OPC: Oropharyngeal cancer (n=14); LC: Laryngeal cancer (n=9); HPC: Hypopharyngeal cancer (n=9); stage II (n=6); stage III (n=4); stage Iva (n=34); stage IVb (n=4); ns: No significance. Source data are provided as a Source Data file.

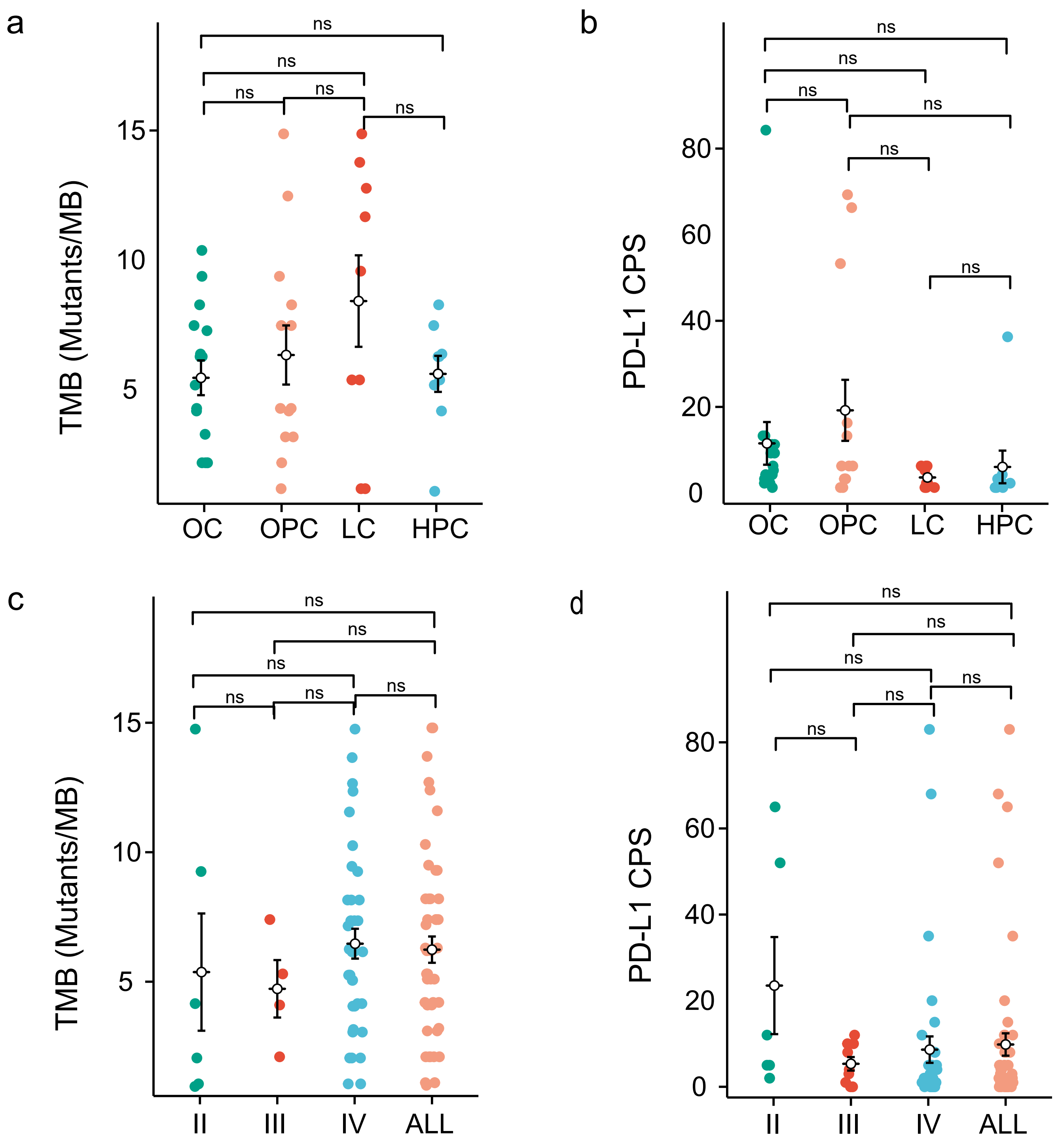

Supplementary Figure 4 Correlation analysis of radiographic tumor response with the density of tumor-infiltrating CD4+ T cells by using the Spearman correlation text (n=38; a). The baby-blue dot represents an individual data point. The line represents a fitted line. The grey shadow represents the corresponding 95% confidence interval. The multiplex immunofluorescence images of the tumor sites in patient 17 (b). Source data are provided as a Source Data file.

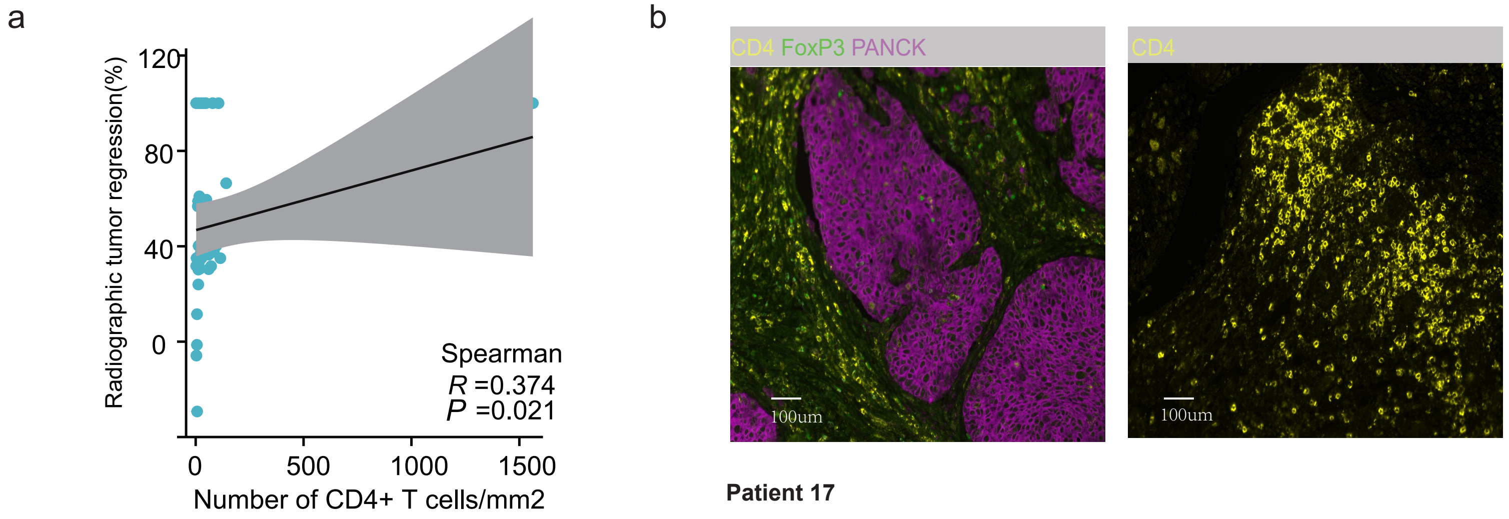

Supplementary Figure 5 Correlation analysis between radiographic tumor regression and TMB (n=47), PD-L1 expression (n=47), or density of tumor-infiltering immune cells (M2-like macrophage cells: n=47; S_M1-like macrophage cells: n=47; S_CD8+ T cells: n=47; S_M2-like macrophage cells: n=47; S_CD56bright cells: n=47; CD56dim cells: n=47; Tregs cells: n=38; S_CD56bright cells: n= 47; S_CD56dim cells: n=47; S_Tregs cells: n=39; S_CD4+ T cells: n=39) by using the Spearman correlation test. The baby-blue dot represents an individual data point. The line represents a fitted line of correlation. The grey shadow represents the corresponding 95% confidence interval. Source data are provided as a Source Data file.

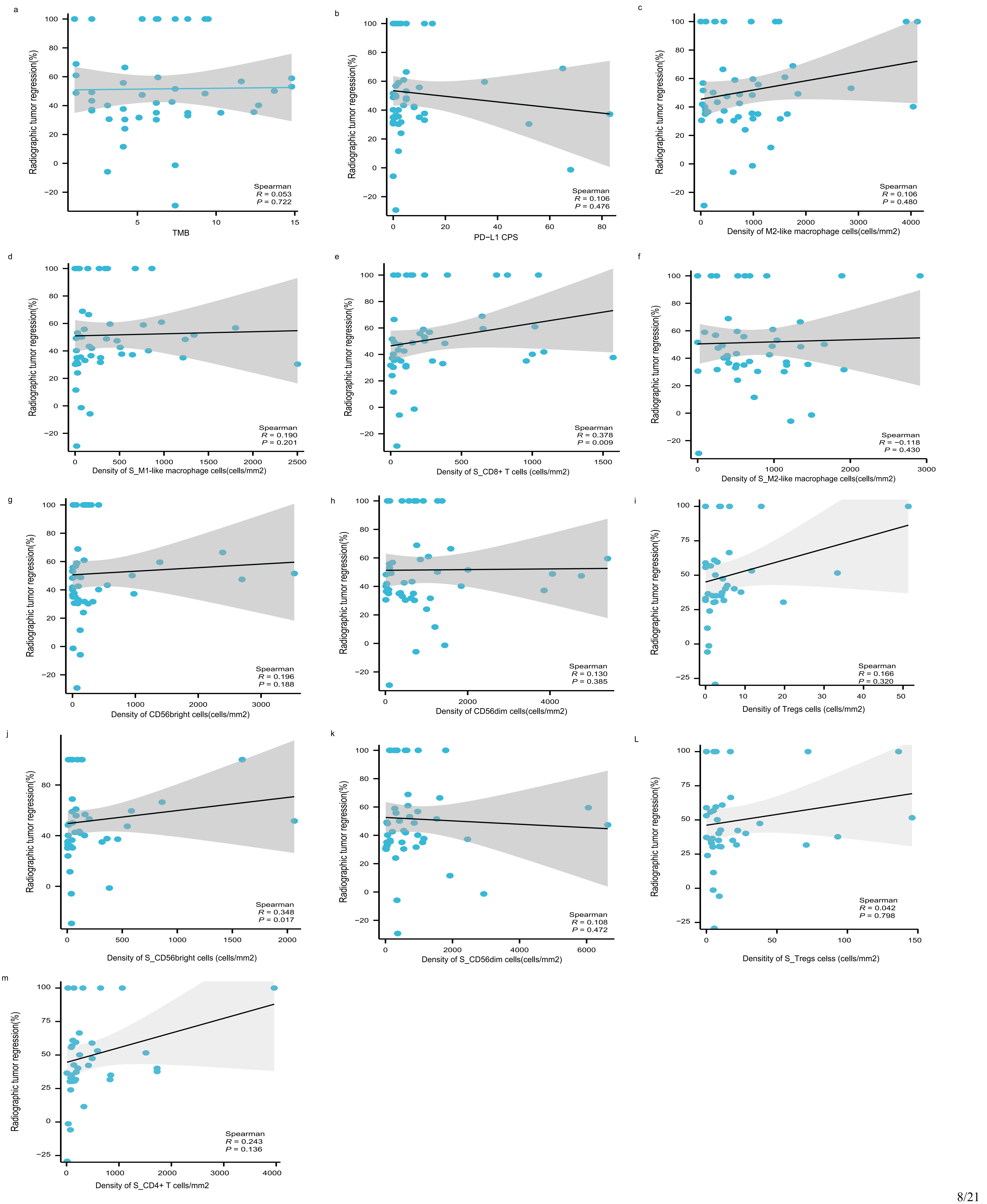

Supplementary Figure 6 Comparisons of the density of tumor infiltration immune cells before and after neoadjuvant therapy in overall patients (n=19, a) and those with pCR (n=8, b) and non-pCR (n=11, c) using the paired Wilcoxon signed-rank test. The blue dot represents an individual data point of cell density at baseline, the red dot represents an individual data point after neoadjuvant therapy. The black line represents the direction of change in cell density following neoadjuvant therapy. *: p<0.05, ns: No significance. Source data are provided as a Source Data file.

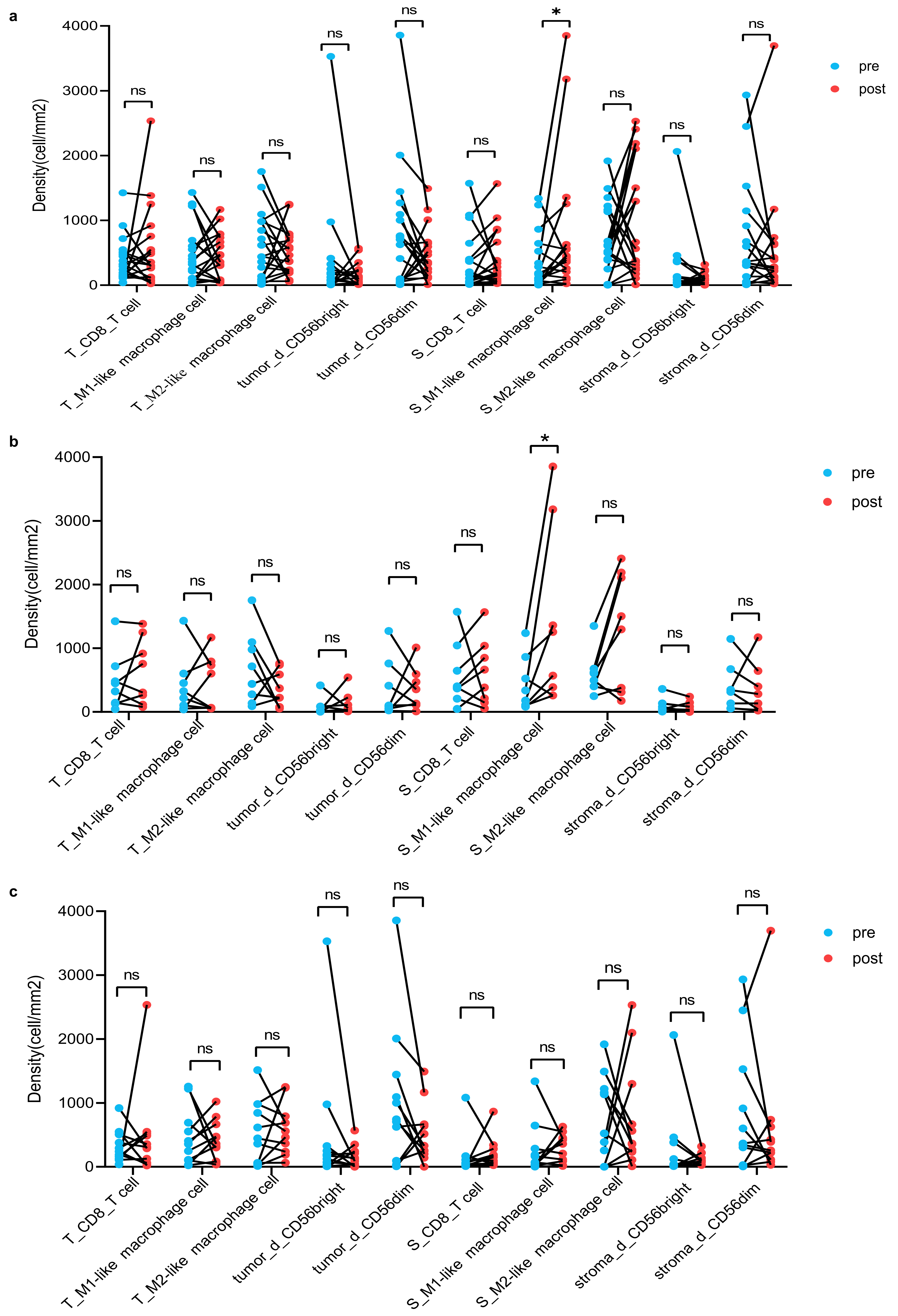

SUPPLEMENTARY NOTE

Neoadjuvant Chemoimmunotherapy with Camrelizumab plus Nab-paclitaxel and Cisplatin (NeoCPC) in Resectable Locally Advanced Squamous Cell Carcinoma of the Head and Neck: A Pilot Phase II Trial

Di Wu[1,#], Yong Li[2,#], Pengfei Xu[1,#], Qi Fang[1], Fei Cao[1], Hongsheng Lin[1], Yin Li[1], Yong Su[3], Lixia Lu[3], Lei Chen[3], Yizhuo Li[4], Zheng zhao[1], Xiaoyu Hong[5], Guohong Li[5], Yaru Tian[6], Jinyun Sun[6], Honghong Yan[7], Yunyun Fan[1], Xinrui Zhang[8],*, Zhiming Li[9,*], Xuekui Liu[1,*]

Corresponding author:

Xuekui Liu

Sun Yat-Sen University Cancer Centre, State Key Laboratory of Oncology in South China, Collaborative Innovation Center for Cancer Medicine, Guangzhou, Guangdong 510060, China

TeL.: +86-020-87343302

E-mail: liuxk@sysucc.org.cn

Zhiming Li

Sun Yat-Sen University Cancer Centre, State Key Laboratory of Oncology in South China, Collaborative Innovation Center for Cancer Medicine, Guangzhou, Guangdong 510060, China.

TeL.: +86-020-87343368

E-mail: lizhm@sysucc.org.cn

Xinrui Zhang

Department of Otolaryngology-Head and Neck Surgery, The Fifth Affiliated Hospital of Guangzhou Medical University, Guangzhou 511436, China

TeL.: +86-020-85959142

E-mail: zxr-850@163.com

Neoadjuvant Chemoimmunotherapy with Camrelizumab plus Nab-paclitaxel and Cisplatin for Resectable Locally Advanced Squamous Cell Carcinoma of the Head and Neck: A Pilot Phase II Trial

Protocol

Project No.: NCT04826679

Version number: Version 2.0

Center:
Sun Yat-Sen University Cancer Centre
State Key Laboratory of Oncology in South China
Collaborative Innovation Center for Cancer Medicine

Principal Investigator:
Xuekui Liu
Dongfeng Road 651, Guangzhou,
Guangdong, 510060, P. R. China.
TeL.: +86-020-87343302
E-mail: liuxk@sysucc.org.cn

Study design:

Single-center, single-arm, opened, phase II trial clinical study.

Intended number of patients:

48 patients.

Patients:

Patients with untreated, resectable, locally advanced (T2–T4, N0–N3b, M0) HNSCC of the oral cavity, oropharynx, hypopharynx, or larynx (stage III–IVb for non-oropharyngeal cancers and HPV-negative oropharyngeal cancer; stage II–III for HPV-positive oropharyngeal cancer, according to the 8th Edition of American Joint Committee on Cancer [AJCC] guideline).

Inclusion criteria:

1. The patient's pathological biopsy results must be confirmed to be squamous cell carcinoma of head and neck by pathologists.
2. Age ≥18 years old and ≤70 years old.
3. ECOG or PS score is 0 or 1.
4. Patients did not previously receive cancer related chemotherapy, radiotherapy, or surgery.
5. Clinical stage should be T2-4/N0-3b/M0 (stage III–IVb for non-oropharyngeal cancers and HPV-negative oropharyngeal cancer; stage II–III for HPV-positive oropharyngeal cancer), with potential of radical surgical treatment.
6. Patients should have PET-CT examination or neck/chest and upper abdomen CT scan, ECT, brain MR/CT, etc. to clarify staging and exclude distant metastasis.
7. Adequate organ and marrow function as defined below:

1) Blood routine examination: Absolute neutrophil count (ANC) $\geq 1.5\times10^9$/L; Platelet count (PLT) $\geq 100\times10^9$/L; Hemoglobin content (HGB) ≥ 9.0g /dL.

2) Liver function: serum total bilirubin (TBIL)≤1.5×Upper Limit of Normal (ULN); Alanine aminotransferase (ALT) and aspartate aminotransferase (AST) ≤2.5×ULN.

3) Renal function: Creatinine clearance (Ccr) ≥60 mL/min (calculated by

Cockcroft/Gault formula).

4) Adequate coagulation function, defined as international normalized ratio (INR) or prothrombin time (PT) ≤1.5 ULN; If patient is receiving anticoagulant therapy, it is acceptable that PT is in the prescribed anticoagulant range.

8. Female patients of reproductive age or male patients whose sexual partner is female patients of reproductive age shall use effective contraceptive measures throughout the treatment period until 6 months after the treatment period.

9. Sign written informed consent and be able to comply with the visits and related procedures specified in the program.

10. Can provide archived pathological tissues or fresh pathological tissues within 6 months for the detection of PD-L1 and other markers.

Exclusion criteria:

1. Patients were taking other investigational drugs at the same time.
2. Any treatment with anti-PD-1, anti-PD-L1, anti-PD-L2, anti-CD137, or anti-CTLA-4 antibodies, or any other antibody or drug that specifically targets at T-cell costimulation or checkpoint pathways prior to study.
3. History of allergic reactions attributed to any monoclonal antibody or chemotherapy drug (paclitaxel, cisplatin) preparations or excipients.
4. Patients were also taking rifampicin, phenytoin sodium, carbamazepine, or barbiturates (these drugs induce CYP3A and may reduce plasma content of paclitaxel).
5. Received systemic therapy of Chinese herbal medicine with anti-tumor indications or immunomodulatory drugs (including thymosin, interferon, interleukin, etc.) within 2 weeks prior to first administration.
6. Administration of a live, attenuated vaccine within 4 weeks prior to the first dose of treatment or planned for it during the study.

Note: Administration of inactivated virus vaccine for seasonal influenza is permitted within 4 weeks prior to the first dose of treatment, while live attenuated flu vaccines are not allowed.

7. Toxicity from prior antineoplastic therapy that did not return to grade 0 or 1 defined

by National Cancer Institute General Adverse Event Terminology version 5.0 (NCI CTCAE version 5.0, excluding alopecia, non-clinically significant or asymptomatic laboratory abnormalities).

8. Known autoimmune disease that needs symptomatic treatment or history of disease within 2 years (patients with vitiligo, psoriasis, hair loss, or graves disease that doesn't need systemic treatment, hypothyroidism that only needs thyroid hormone replacement therapy and type 1 diabetes which only need insulin replacement therapy can be enrolled).

9. Known history of primary immunodeficiency.

10. Known to have active infection of tuberculosis.

11. Known history of allogeneic organ transplantation and allogeneic hematopoietic stem cell transplantation.

12. HIV infection and carriers are known to exist (HIV antibody positive).

13. Severe infections that are in active period or clinically poorly controlled.

14. Symptomatic congestive heart failure (NYHA II-IV) or symptomatic or poorly controlled arrhythmia.

15. Uncontrolled arterial hypertension (systolic blood pressure ≥160mmHg or diastolic blood pressure ≥100mmHg) even with standard therapy.

16. Any arterial thromboembolic event, including myocardial infarction, unstable angina, cardiocerebral events or transient ischemic attack (TIA), occurred within 6 months prior to enrollment.

17. Significant malnutrition which need intravenous supplements while malnutrition corrected for over 4 weeks prior to first dose of the study was excluded.

18. A history of deep vein thrombosis, pulmonary embolism, or any other severe thromboembolism within 3 months prior to enrollment (Implantable Venous Access Port or duct-derived thrombosis, or superficial venous thrombosis is not considered as "severe" thromboembolism).

19. Uncontrolled metabolic disorders or other non-malignant organ or systemic diseases or secondary reactions to cancer that may result in higher medical risk and/or uncertainty in the assessment of survival.

20. Hepatic encephalopathy, hepatorenal syndrome, Child-Pugh B liver cirrhosis or worse.

21. History of intestinal obstruction or following diseases: inflammatory bowel disease (IBD) or extensive bowel resection (partial resection of the colon or extensive resection of the small intestine with chronic diarrhea), Crohn's disease, ulcerative colitis (UC).

22. Known to have acute or chronic active hepatitis B (HBsAg positive with HBV DNA viral load $\geq 10^3$ IU/ml /mL or >200IU/ mL) or acute or chronic active hepatitis C (HCV antibody positive and HCV RNA positive).

23. History of gastrointestinal perforation and/or fistula within 6 months prior to enrollment.

24. Interstitial lung disease (ILD) requiring steroid therapy.

25. History of other primary malignancies.

26. Pregnant or lactating female patients.

27. Other acute or chronic diseases, psychiatric disorders, or abnormal laboratory test values that may cause: increase related risk of study participation or drug administration, interfere with the interpretation of study results or ineligible patients for study participation from researcher's judgment.

28. Patients with difficulty in obtaining satisfactory biopsy specimens.

Withdraw criteria:

1. The patient is found to be ineligible for the inclusion/exclusion criteria and is deemed unsuitable for further study by the investigator.

2. The patient violates the study protocol, and it is considered inappropriate to continue to participate in further study by the investigator.

3. Any other type of pharmaceutical research that is considered scientifically or medically incompatible with this study.

4. The patient fails to finish the defined follow-up evaluations (Research center staff should contact the patient who has lost follow-up in order to determine the reason and try to reschedule the visit. The date the patient was contacted, and the contact details should be recorded in the study file).

5. Evaluated by investigator

1) If, for any reason, the patient needs to be treated with another drug that has been shown to be effective in treating the indication of the study, the patient should withdraw from the study before using the new drug.

2) Any treatment-related event considered life-threatening has occurred,regardless of the severity of the event.

3) Study drugs meet withdrawal criteria due to toxicity.

4) Patient or client (such as parent or legal guardian) requests to withdraw from the study or discontinuation of study drugs (if patient withdraws informed consent for treatment but not for follow-up, long-term followup is still available.

5) The Investigator or co-sponsors may terminate the study or discontinue the patient's participation for medical, safety, regulatory, or other reasons related to GCP.

Drugs, Dose, Route, Regimen:

(1) Camrelizumab

- Specification: 200 mg/ bottle
- Administration method: 200mg IV D1 Q3W

(2) Nab-paclitaxel

- Specification: 100 mg/ piece
- Administration method: 260mg/m2 IV D1 Q3W

(3) Cisplatin

- Specification: 30 mg/ piece
- Administration method: 60mg/m2 IV D1 Q3W

Primary endpoint:

The primary endpoint was the objective response rate (ORR) defined as the proportion of patients achieving CR or PR.

Secondary endpoints:

Secondary endpoints included pathological complete response (pCR), major

pathological response (MPR), two-year progression-free survival (PFS) rate, two-year OS rate, and toxicities.

Exploratory endpoints：

Genomic biomarkers (genetic mutations, tumour mutational burden [TMB] and immune microenvironment) in baseline tumour samples were investigated.

Follow-up duration：

Two-year follow-up.

Evaluation Criteria:

1. Safety evaluation:

The incidence and severity of all Adverse Events (AE), Treatment Emergent Adverse Even (TEAE), Adverse Events of Special Interest (AESI), and Serious Adverse Events (SAE) were evaluated according to CTCAE version 5.0.

The changes of vital signs, physical examination results, and laboratory results before, during, and after treatment.

Assessment of surgical safety: surgical R0 resection rate, operation duration, blood loss, length of hospital stay, incidence of complications, perioperative mortality, etc.

2. Effectiveness evaluation:

The ORR was assessed according to RECIST1.1 criteria. Pathological evaluation: MPR rate, pCR rate, DCR rate, R0 resection rate, tumor infiltrating lymphocytes (TIL), tumor cell death rate, etc. Follow-up of survival: Progression-free survival (PFS) and Overall Survival(OS).

(3) Biomarkers:

Tumor tissue samples were collected for tumor biomarker analysis, including but not limited to the changes of the following indicators in tumor specimens (PD-L1, HPV infection, TMB, TAMs etc.).

Study Duration:

The patients were enrolled from April 2021 to March 2022, and patients were followed up for long-term survival after the intervention.

Trial Progress:

1. Patients need to complete relevant examinations and evaluations. If the patients meet all inclusion and exclusion criteria, they will be enrolled and sign the informed consent.
2. Enrolled patients will receive 3 cycles of camrelizumab (200mg IV D1 Q3W) combined with cisplatin (60 mg/m^2, IV D1 Q3W) and nab-paclitaxel (260mg/m^2, IV D1 Q3W), and its safety was evaluated during the treatment.
3. After completing 3 cycles of camrelizumab combined with cisplatin and albumin-paclitaxel, the patient needs to return to the hospital for re-examination of physical examination, blood biochemical examination, liver and kidney function examination. Neck and chest CT scan needs to be performed again. Patients suspected of distant metastasis should be performed examination of the specific site to confirm the diagnosis. At the same time, relevant preoperative examination should be performed to exclude surgical contraindications. Efficacy was evaluated according to RECIST 1.1 criteria.
4. Surgical treatment: the surgical method adopted in this study was radical resection of head and neck cancer. The primary specimens and enlarged lymph nodes were obtained at baseline and at the time of surgery. Surgery is performed within 3-6 weeks after completion of the third chemotherapy. Surgical margin: the proximal and distal margin should be over 1 cm beyond the edge of tumor. Pathology was used to determine the depth of tumor invasion, whether the resection margin contained tumor cells, and the proportion of tumor cells. This procedure is used to assess R0 resection, pathological response rate, and subsequent treatment decisions.
5. Radiotherapy: Patients with laryngeal or hypopharyngeal cancer whose disease responded well to NeoCPC (complete response [CR] or partial response [PR] per RECIST version 1.1) received (chemo)radiotherapy directly or following cervical lymph node dissection to preserve organ function.
6. After treatment, regular follow-up of survival was performed every 3 months in the

first year after surgery, and every 6 months in 2-5 years after surgery, including recurrence and related treatment.

Statistical analysis plan：

This study used Simon's two-stage design. The null hypothesis p0 was set at an ORR rate of ≤72% while the alternative hypothesis was an ORR rate of ≥88%. Assuming a two-sided α value of 0.05 and a power (1-β) of 80%, 11 patients were required to be enrolled in the first stage. If fewer than 8 patients achieved a CR or PR, the trial would be permanently stopped. Otherwise, the trial would continue to enroll an additional 36 patients in the second stage. The null hypothesis would be rejected if 39 or more patients achieved a CR or PR.

The binary endpoints (ORR and pCR) were expressed as frequency and percentage and the corresponding 95% confidence intervals (CIs) were estimated by using the normal approximation method. The correlation of potential biomarkers (PD-L1, HPV infection, immune infiltration immune cell subsets) with the radiographic and pathological response was estimated by using Spearman's rank correlation coefficient. The correlation between PD-L1 expression and pathological response was evaluated by using the Fisher exact test. Kaplan-Meier method was used to estimate PFS and OS. Safety was summarized in treated patients using descriptive statistics.

Oncoplots, constructed by R (3.5.2), was used to view the overall mutation landscape of HNSCC patients. Fisher's exact test was used to compare the proportions between groups, and the OR with 95% CI for response to treatment was provided. A two-sided $p<0.05$ was considered significant for all tests unless otherwise indicated. All statistical analyses were performed by using the R V3.5.2.

Safety:

According to the mechanism of camrelizumab and clinical safety information of products with the same mechanism, adverse events may occur in the process of this clinical trial are primarily reactive capillary hyperplasia and immunity inflammation

caused by the immune system activation such as pneumonia, colitis, hepatitis, nephritis, renal insufficiency and inflammation of the endocrine system, etc. According to the clinical data of existing anti-PD-1 monoclonal antibody drugs, although the incidence of adverse events is high, however these are tolerated. For early symptoms of immune related adverse reaction are varied, special attention should be paid to early signs and symptoms of various immune related reaction to make correct judgement in time and perform dose adjustment according to the plan and give the specific treatment. At the same time, attention should be paid to exclude patients with autoimmune diseases to avoid exacerbation of the original disease caused by activation of the immune system.
