# Supplementary material for: Neoadjuvant chemo-immunotherapy with camrelizumab plus nab-paclitaxel and cisplatin in resectable locally advanced squamous cell carcinoma of the head and neck: a pilot phase II trial
Source: Nat Commun. 2024 Mar 11;15:2177. doi: 10.1038/s41467-024-46444-z (PMC10928200; doi:10.1038/s41467-024-46444-z)
Supplement: Supplementary file 3 — Reporting Summary [file 41467_2024_46444_MOESM3_ESM.pdf]

nature portfolio

Corresponding author(s): Xuekui Liu
Last updated by author(s): Jan 8, 2024

Reporting Summary

Nature Portfolio wishes to improve the reproducibility of the work that we publish. This form provides structure for consistency and transparency in reporting. For further information on Nature Portfolio policies, see our Editorial Policies and the Editorial Policy Checklist.

Please do not complete any field with "not applicable" or n/a. Refer to the help text for what text to use if an item is not relevant to your study.
For final submission: please carefully check your responses for accuracy; you will not be able to make changes later.

Statistics

For all statistical analyses, confirm that the following items are present in the figure legend, table legend, main text, or Methods section.

n/a	Confirmed	
☐	☒	The exact sample size (n) for each experimental group/condition, given as a discrete number and unit of measurement
☐	☒	A statement on whether measurements were taken from distinct samples or whether the same sample was measured repeatedly
☐	☒	The statistical test(s) used AND whether they are one- or two-sided<br>*Only common tests should be described solely by name; describe more complex techniques in the Methods section.*
☐	☒	A description of all covariates tested
☒	☐	A description of any assumptions or corrections, such as tests of normality and adjustment for multiple comparisons
☐	☒	A full description of the statistical parameters including central tendency (e.g. means) or other basic estimates (e.g. regression coefficient) AND variation (e.g. standard deviation) or associated estimates of uncertainty (e.g. confidence intervals)
☐	☒	For null hypothesis testing, the test statistic (e.g. F, t, r) with confidence intervals, effect sizes, degrees of freedom and P value noted<br>*Give P values as exact values whenever suitable.*
☒	☐	For Bayesian analysis, information on the choice of priors and Markov chain Monte Carlo settings
☒	☐	For hierarchical and complex designs, identification of the appropriate level for tests and full reporting of outcomes
☐	☒	Estimates of effect sizes (e.g. Cohen's d, Pearson's r), indicating how they were calculated

Our web collection on statistics for biologists contains articles on many of the points above.

Software and code

Policy information about availability of computer code

Data collection	Microsoft Excel was used for data collection.
Data analysis	Microsoft Excel and Graphpad Prism 9.0 R(4.5.3) were used for data analysis and figure generation.

For manuscripts utilizing custom algorithms or software that are central to the research but not yet described in published literature, software must be made available to editors and reviewers. We strongly encourage code deposition in a community repository (e.g. GitHub). See the Nature Portfolio guidelines for submitting code & software for further information.

Data

Policy information about availability of data

All manuscripts must include a data availability statement. This statement should provide the following information, where applicable:

- Accession codes, unique identifiers, or web links for publicly available datasets
- A description of any restrictions on data availability
- For clinical datasets or third party data, please ensure that the statement adheres to our policy

The study protocol is available as Supplementary Note in the Supplementary Information file. All raw targeted DNA-sequencing data have been deposited in the National Genomics Data Center (NGDC) under the accession code HRA005542. The deposited and publicly available data are compliant with the regulations of the Ministry of Science and Technology of the People’s Republic of China. The raw sequencing data contain information unique to individuals are available under controlled access. Access to the data can be requested by completing the application form via GSA-Human System and is granted by the corresponding Data Access

Committee. Additional guidance can be found at the GSA-Human System website [https://ngdc.cncb.ac.cn/gsa-human/document/GSA-Human_Request_Guide_for_Users_us.pdf]. The raw patient data are not available due to the data privacy laws. The de-identified individual patient data will be available upon reasonable request for academic research purposes by contacting the corresponding author (liuxk@sysucc.org.cn) for 10 years. The remaining data are available within the Article, Supplementary Information, and Source Data file. Source data are provided with this paper.

Research involving human participants, their data, or biological material

Policy information about studies with human participants or human data. See also policy information about sex, gender (identity/presentation), and sexual orientation and race, ethnicity and racism.

Reporting on sex and gender	The research findings apply to both sexes. Both men and women can develop head and neck squamous cell carcinoma. There was no restriction on sex of patients eligible for inclusion. Only six patients were female, and thus no post hoc sex based analysis was performed.
Reporting on race, ethnicity, or other socially relevant groupings	All patients enrolled were Han Chinese. No reporting on race, ethnicity, or other socially relevant groupings are applicable.
Population characteristics	Patients with untreated, resectable, locally advanced (T2–T4, N0–N3b, M0) HNSCC of the oral cavity, oropharynx, hypopharynx, or larynx (stage III IVb for non oropharyngeal cancers and HPV negative oropharyngeal cancer; stage II III for HPV positive oropharyngeal cancer, according to the 8th Edition of American Joint Committee on Cancer [AJCC] guideline.
Recruitment	Consecutive patients with resectable HNSCC for the study were offered for potential participation in the clinical trial at Sun Yat Sen University Cancer Centre. Eligible patients were 18 years or older, previously untreated, resectable, locally advanced HNSCC. This was a single-arm, open label, phase II clinical trial. There was no self-selection bias and other biases.
Ethics oversight	The clinical trial was approved by the research ethics board of Sun Yat Sen University Cancer Centre (Guangzhou, China).

Note that full information on the approval of the study protocol must also be provided in the manuscript.

Field-specific reporting

Please select the one below that is the best fit for your research. If you are not sure, read the appropriate sections before making your selection.

☒ Life sciences ☐ Behavioural & social sciences ☐ Ecological, evolutionary & environmental sciences

For a reference copy of the document with all sections, see nature.com/documents/nr-reporting-summary-flat.pdf

Life sciences study design

All studies must disclose on these points even when the disclosure is negative.

Sample size	This study used Simon's two-stage design. The null hypothesis p0 was set at an ORR rate of 72%, while the alternative hypothesis was an ORR rate of 88%. Assuming a two-sided α value of 0.05 and a power (1-β) of 80%, 11 patients were required to be enrolled in the first stage. If fewer than eight patients achieved a CR or PR, the trial would be permanently stopped. Otherwise, the trial would continue to enroll an additional 36 patients in the second stage. The null hypothesis could be rejected if 39 or more patients achieved a CR or PR.
Data exclusions	Patients that signed the informed consent but did not receive any treatment were excluded from the analysis.
Replication	Not applicable. This is a single arm phase II clinical trial, involving assessment of the efficacy and safety of neoadjuvant chemotherapy plus immune checkpoint inhibitors in human participants. No preclinical data that could be replicated are provided in the manuscript.
Randomization	Not applicable. This was a pilot single-arm phase II trial to explore the efficacy and safety of neoadjuvant camrelizumab plus chemotherapy
Blinding	Not applicable. This was an open label clinical trial. Given that there was no randomization, and patients were included in one consecutive cohort, the blinding did not apply.

Reporting for specific materials, systems and methods

We require information from authors about some types of materials, experimental systems and methods used in many studies. Here, indicate whether each material, system or method listed is relevant to your study. If you are not sure if a list item applies to your research, read the appropriate section before selecting a response.

Materials & experimental systems

n/a	Involved in the study
☐	☒ Antibodies
☒	☐ Eukaryotic cell lines
☒	☐ Palaeontology and archaeology
☒	☐ Animals and other organisms
☐	☒ Clinical data
☒	☐ Dual use research of concern
☒	☐ Plants

Methods

n/a	Involved in the study
☒	☐ ChIP-seq
☒	☐ Flow cytometry
☒	☐ MRI-based neuroimaging

Antibodies

Antibodies used

PANO 7 plex IHC kit (cat 0004100100; Panovue, Beijing, China). PANO 6 plex IHC kit (TSA RM 82758, Panovue, Beijing, China). PD L1 expression was assessed by using the 22C3 pharmDx assay (1:50, Dako, M3653) on the DAKO Autostainer Link 48 platform.

Validation

PANO 7 plex IHC kit and PANO 6 plex IHC kit are commercially manufactured and marketed kits that are widely used in immune micro environment test. The PD L1 IHC 22C3 pharmDx kit is a FDA approved IHC companion in vitro diagnostic assay.

Clinical data

Policy information about clinical studies

All manuscripts should comply with the ICMJE guidelines for publication of clinical research and a completed CONSORT checklist must be included with all submissions.

Clinical trial registration

NCT04826679

Study protocol

The full clinical trial protocol has been uploaded as supplementary note.

Data collection

Data was collected using Microsoft Excel at Sun Yat Sen University Cancer Centre.
Between April 19, 2021 and March 17, 2022, a total of 48 patients were enrolled. The data collection started with the enrollment of the first patient on April 2021 and the follow up is ongoing.

Outcomes

The primary endpoint was the objective response rate (defined as the proportion of patients achieving CR or PR per RECIST v1.1. Secondary endpoints included pathological complete response (pCR), major pathological response (MPR), two year progression free survival (PFS) rate, two year OS rate, and toxicities. The radiographic assessment was performed at baseline, nine weeks after treatment initiation, and every six months thereafter, according to the RECIST v1.1. The primary specimens and enlarged lymph nodes were obtained at baseline and at the time of surgery. Pathologic response was determined by senior pathologists who were blinded to the clinical data of the patients. Both the primary tumors and lymph nodes were used to assess pathologic response.

Plants

Seed stocks

Not applicable

Novel plant genotypes

Not applicable

Authentication

Not applicable

This checklist template is licensed under a Creative Commons Attribution 4.0 International License, which permits use, sharing, adaptation, distribution and reproduction in any medium or format, as long as you give appropriate credit to the original author(s) and the source, provide a link to the Creative Commons license, and indicate if changes were made. The images or other third party material in this article are included in the article's Creative Commons license, unless indicated otherwise in a credit line to the material. If material is not included in the article's Creative Commons license and your intended use is not permitted by statutory regulation or exceeds the permitted use, you will need to obtain permission directly from the copyright holder. To view a copy of this license, visit http://creativecommons.org/licenses/by/4.0/
